# Is esketamine-based opioid-free anesthesia more superior for postoperative analgesia in obstructive sleep apnea patients undergoing bariatric surgery? A study protocol

**DOI:** 10.3389/fmed.2022.1039042

**Published:** 2022-11-15

**Authors:** Yongle Guo, Lina Chen, Zhongquan Gao, Min Zhang, Mengjie Liu, Xiaojun Gao, Yang Liu, Xiaoning Zhang, Na Guo, Yongtao Sun, Yuelan Wang

**Affiliations:** ^1^Department of Anesthesiology, The First Affiliated Hospital of Shandong First Medical University & Shandong Provincial Qianfoshan Hospital, Shandong Institute of Anesthesia and Respiratory Critical Medicine, Jinan, China; ^2^Department of Anesthesiology, Shandong First Medical University, Jinan, China

**Keywords:** esketamine, opioid-free anesthesia, postoperative analgesia, OSA, bariatric surgery

## Abstract

**Introduction:**

Opioid-free anesthesia (OFA) can certainly prevent nausea and vomiting after bariatric surgery (BS), but its postoperative analgesic effect is still controversial. Obstructive sleep apnea (OSA) is a prominent feature of morbid obesity in BS and accounts for a very high proportion, which significantly increases the difficulty of patients’ airway management. Those patients will be more representative and highlight the advantages of OFA. It is not clear whether esketamine can play a more prominent role in OFA for postoperative analgesia. Therefore, this study aims to explore the postoperative analgesic effect of esketamine-based OFA on BS patients with OSA.

**Methods and analysis:**

This single-center, prospective, randomized, controlled, single-blind study is planned to recruit 48 participants to undergo BS from May 2022 to April 2023. Patients will be randomly assigned to the OFA group and opioid-based anesthesia (OBA) group in a ratio of 1:1. The primary outcome is the Numeric Rating Scale (NRS) at different times postoperatively. Secondary outcomes include analgesic intake, the incidence and severity of postoperative nausea and vomiting (PONV), Leiden Surgical Rating Scale (L-SRS), postoperative agitation and chills, PACU stay time, EuroQol five-dimensional questionnaire (EQ-5D), length of hospital stay, intraoperative awareness, and hemodynamically unstable treatments.

**Discussion:**

The results of this study may explain the analgesic effect of esketamine-based OFA on patients undergoing BS combined with OSA, and provide evidence and insight for perioperative pain management.

**Ethics and dissemination:**

This study is initiated by the Ethics Committee of The First Affiliated Hospital of Shandong First Medical University [YXLL-KY-2022(035)]. The trial results will be published in peer-reviewed journals and at conferences.

**Clinical trial registration:**

[https://clinicaltrials.gov/ct2/show/NCT05386979], identifier [NCT 05386979].

## Background

About 500,000 people worldwide have undergone BS surgery, and the number continues to grow by 2015 (1). Morbid obesity is associated with multiple comorbidities, the most common of which is OSA. OSA is present in 35–94% of morbid obesity patients (2–7). Morbid obesity and OSA are often associated with increased perioperative risks and challenges for anesthesiologists (8). Risks conferred by OSA are strongly associated with body mass index (BMI) (9, 10). One study showed a 6-fold increased risk of OSA with 10% weight gain (11). Another study showed that the prevalence of moderate to severe OSA (AHI > 15) was 63% in obese males (BMI > 30 kg/m^2^) (12). Morbid obesity is a leading cause of early mortality worldwide, and currently, BS remains the only proven effective and durable therapy. Obese patients undergoing BS have a high probability of developing complications that worsen with opioid use but can be reduced by anesthetic techniques such as OFA (13).

Opioids have long been established as essential for general anesthesia, and in all patients, opioids induce and increase the severity of most sleep-disordered breathing, especially in patients with morbid obesity. OFA shows evidence of its efficacy and safety while its risks and benefits are not well-defined. However, opioid-induced hyperalgesia and tolerance further drive the use of intraoperative opioid-sparing strategies based on a combination of regional nerve block techniques or other anesthetic technical means (14, 15). Crivits et al. (16) reported those who received OFA compared with those who received sufentanil anesthesia had significantly less nausea, cold, shivering or pain in an observational study of 400 patients undergoing laparoscopic gastric bypass. The definition of OFA is varied in literature and in research. However, lidocaine, ketamine, and α-2 agonists (e.g., clonidine or dexmedetomidine) have been proposed to be used alone or in combination to replace opioids (17). Studies show that ketamine has been used as one of the well-established drugs for OFA (18–20). However, ketamine’s side effects, including nightmares and delusions, limit its routine use (20, 21).

The analgesic effect of esketamine, the S (+)-isomer of ketamine, is twice of racemic ketamine. Esketamine possesses advantages of a lower incidence of side effects like hallucinations, faster recovery, and the ability to lower MAC value of sevoflurane as well as protect hypoxic pulmonary. Ketamine has been suggested to be used alone or in combination with opioids. Esketamine has long been considered an effective treatment for depression. Currently, it shows that esketamine is effective against remifentanil-induced respiratory depression, which is attributed to increased CO_2_ chemosensitivity by esketamine. However, whether esketamine can replace ketamine in playing a more prominent role in the OFA remains unclear. Therefore, this study is designed to investigate the effects of esketamine-based OFA on the analgesic management of patients undergoing BS with OSA.

## Methods and analysis

### Trial objectives and study design

**FIGURE 1:**
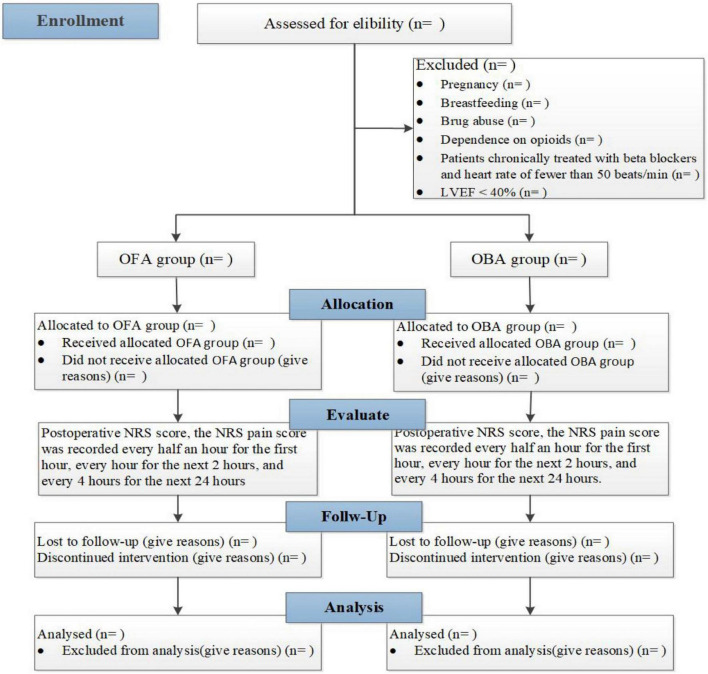
General review of study design (flow diagram).

This single-center, prospective, randomized, controlled and single-blind study will be performed at the First Affiliated Hospital of Shandong First Medical University, located in Jinan City, Shandong Province. Patients will be assigned to receive OFA or OBA randomly. We will evaluate the pain management in randomized morbid obesity patients with OSA undergoing BS by a Numeric Rating Scale (NRS score) in the time points at different times within 27 h after the operation. This trial will be completed in 12 months. This trial is designed following the Standard Protocol Items (SPIRIT guidelines). Figure 1 and Table 2 provide an overview of the study plan.

### Randomization and blinding

Patients will be randomized using block randomization with random block length, stratified to minimize bias on the primary outcome measure. Randomization will be performed electronically after the assessment of eligibility. The patients and surgical staff will be blinded to the group allocation in this study, whereas, anesthesia providers who did not participate in the assessment of the patients at any time could not be blinded to facilitate intraoperative anesthesia management. A blinded independent researcher will be responsible for preoperative visit and obtaining informed consent with patients. The outcome will be evaluated by this independent researcher to minimize the bias associated with data collection. The statisticians will also be blinded to the allocation.

### Participants’ inclusion and exclusion criteria

During the anesthesia consultation, investigators will verify inclusion/exclusion criteria. The investigator will invite the patients to participate. Patients will receive complete information in faithful terms and understandable language concerning the objectives, the required follow-up, the risks, the safety measures, and the right to refuse to participate or stop the study at any time. The investigator will obtain written informed consents signed by both the investigator and the patient.

#### Inclusion criteria

1. Age 18–60 years old.
2. ASA I∼III level.
3. BMI > 35 kg/m^2^.
4. Bariatric surgery for patients with moderate to severe OSA.

#### Exclusion criteria

1. Pregnancy or breastfeeding.
2. Patients with a history of drug abuse or dependence on opioids.
3. Patients chronically treated with beta-blockers and heart rate of fewer than 50 beats/min.
4. Cardiac insufficiency with a left ventricular ejection fraction of less than 40%.

#### Shedding criteria

1. Reoperation during the observation period.
2. Unconsciousness or mortality during the observation period.
3. Discharge automatically or transferred in advance.
4. The patient or the client refuses the informed consent or requests to withdraw from the study during the observation period.

### Intervention

The study aims to compare the OFA protocol with a standard practice-based anesthesia protocol. Patients will be divided into two groups according to the randomization method described later in a ratio of 1:1 in group OFA or OBA. Patients will receive general anesthesia combined with regional anesthesia. The two protocols are detailed in Table 1.

**TABLE 1:** Detailed interventional protocols in the opioid-free anesthesia group (OFA group) and opioid-based anesthesia group (OBA group).

Opioid-free anesthesia protocol	Opioid-based anesthesia protocol

Before surgery	
● IV: midazolam 2 mg, atropine 0.4 mg ● The patient’s position: head-high slope ● Bilateral recurrent laryngeal nerve block (0.375% ropivacaine 2 ml on each side) under ultrasound guidance ● Dyclonine mucilage 10 ml mouth will be contained 5 min ● Dexmedetomidine loading capacity: 1 μg/kg/10 min ● Use a laryngeal tube to test the feeling of the back of the oropharynx, if there is a nausea reflex, add 2% lidocaine 2 ml. ● Insert Video LMA SACOVLM*™* while awake ● Induction of anesthesia once vocal cords will be visible and end-tidal carbon dioxide waveform is observed	

**Anesthesia induction**	

● **Propofol** 2.5 mg/kg ● **Esketamine** 0.5 mg/kg ● **Rocuronium** 0.6 mg/kg ● **Nalbuphine** 10 mg ● Subcostal ultrasound-guided bilateral transversus abdominis plane block: 0.375% ropivacaine 40 ml	● **Propofol** 2.5 mg/kg ● **Sufentanil** 0.3 μg/kg ● **Rocuronium** 0.6 mg/kg ● **Nalbuphine** 10 mg ● Subcostal ultrasound-guided bilateral transversus abdominis plane block: 0.375% ropivacaine 40 ml

**Anesthesia maintenance**	

● **Propofol TCI Ce** 2–4 μg/ml ● **Esketamine** 0.2–0.5 mg/kg/h ● **Dexmedetomidine** 0.5–2 μg/kg/h ● **Esmolol** 20–50 μg/kg/min ● **Rocuronium** 0.6 mg/kg ● **Nalbuphine** 10 mg	● **Propofol TCI Ce** 2–4 μg/ml ● **Remifentanil TCI Ce** 3–6 ng/ml ● **Dexmedetomidine** 0.5–2 μg/kg/h ● **Sufentanil** 10 μg after surgery ● **Rocuronium** 0.6 mg/kg ● **Nalbuphine** 10 mg

**Postanesthesia care unit (PACU)**	

● **Sugammadex** 2 mg/kg ● **Pain management** (VAS ≥ 4, rescue nalbuphine 5 mg) ● **PCIA** nalbuphine 2 mg/kg + dexmedetomidine 2 μg/kg + ondansetron 24 mg	● **Sugammadex** 2 mg/kg ● **Pain management** (VAS ≥ 4, rescue nalbuphine 5 mg) ● **PCIA** sufentanil 2 μg/kg + dexmedetomidine 2 μg/kg + ondansetron 24 mg

LMA SACOVLM™, Zhejiang UE Medical Corp (Hangzhou, China). IV, intravenous; TCI, target controlled infusion; PACU, postanesthesia care unit; PCIA, patient-controlled intravenous analgesia.

**TABLE 2:** Study timeline and schedule of enrolment, allocation, interventions, and assessments according to SPIRIT 2013 statement.

	Study period														
	Enrolment	Allocation	During surgery	Post-operation											Close-out
			
Timepoint	−D_1_	−D_1_	0	PACU	0.5 h	1 h	2 h	3 h	7 h	11 h	15 h	19 h	23 h	27 h	D_7_
Enrolment:															
Eligibility screen	X														
Informed consent	X														
Allocation		X													
Interventions:															
[OFA group]		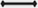													
[OBA group]		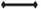													
Assessments:															
[Inclusion/exclusion criteria]	X	X													
[Baseline data]	X	X													
[L-SRS]			X												
[Postoperative agitation]				X											
[Postoperative chills]				X											
[Length of stays]				X											X
[Rescue antiemetic medication]				X	X	X	X	X	X	X	X	X	X	X	
[Vital signs]			X	X											
[NRS score]				X	X	X	X	X	X	X	X	X	X	X	
[Incidence and severity of PONV]				X	X	X	X	X	X	X	X	X	X	X	
[Intraoperative awareness]				X	X	X	X	X	X	X	X	X	X	X	
[EQ-5D]															X

In group OFA, anesthesia induction with propofol 2.5 mg/kg, rocuronium bromide 0.6 mg/kg, nalbuphine 10 mg, esketamine 0.5 mg/kg, intubation will be performed when BIS reached 40–60, followed by a continuous intravenous infusion of propofol TCI Ce 2–4 μg/ml and esketamine 0.2–0.5 mg/kg/h and esmolol 20–50 μg/kg/min. Nalbuphine 10 mg will be given at the beginning of the operation. Ondansetron 8 mg and nalbuphine 0.2 mg/kg will be given before abdominal suturing. After the operation, a PCIA will be used (nalbuphine 2 mg/kg + dexmedetomidine 2 μg/kg + ondansetron 24 mg) in a total volume of 100 ml and continuous infusion at a rate of 1.5 ml/h for 48 h. The self-controlled capacity is 0.5 ml, and the locking time is 15 min.

In group OBA, anesthesia induction with propofol 2.5 mg/kg, rocuronium bromide 0.6 mg/kg, nalbuphine 10 mg, sufentanil 0.3 μg/kg, intubation will be performed when BIS reached 40–60 and followed by a continuous intravenous infusion of propofol TCI Ce 2–4 μg/mL and remifentanil TCI Ce 3–6 ng/ml. Nalbuphine 10 mg will be given at the beginning of the operation. Ondansetron 8 mg and sufentanil 10 μg will be given before abdominal suturing. Patients will be equipped with a PCIA (sufentanil 2 μg/kg + dexmedetomidine 2 μg/kg + ondansetron 24 mg, total volume 100 ml, 1.5 ml/h for 48 h). The self-controlled capacity is 0.5 ml, and the locking time is 15 min.

### Monitoring and standard practice-based anesthesia protocol

All patients will not receive premedication. After admission to the operating room, the participants are placed in the slope position and will be continuously monitored using ECG, pulse oxygen saturation, end-tidal carbon dioxide concentration, non-invasive blood pressure, and the bispectral index (BIS) of electroencephalography (EEG). Radial artery catheterization will be performed to monitor invasive blood pressure, subsequently, midazolam 2 mg and atropine 0.4 mg were administered intravenously.

We chose the method of endotracheal intubation under a visual laryngeal mask to control the airway. All patients will be regarded as having difficult airway and placed the visual laryngeal mask in the conscious state, ultrasound-guided bilateral recurrent laryngeal nerve block will be injected with 0.375% ropivacaine 2 ml, respectively. Dacronin 10 ml Contained in the mouth for about 5 min. Dexmedetomidine (load capacity 1 μg/kg/10 min maintenance dose 0.6 μg/kg/h until 40 min before the end of the operation) will be injected with a micromedicine infusion pump. The model of laryngeal mask was selected according to the patient’s lean weight and 100% oxygen will be delivered after the anesthesia circuit connected. Endotracheal intubation will be performed once the vocal cord and PetCO_2_ waveform were seen, and it will be used to maintain anesthesia during the operation with the laryngeal mask cuff gas evacuated retained. Both groups will be combined with ultrasound-guided transversus abdominis plane block (with 0.375% ropivacaine 40 ml).

Patients will enter different groups based on the results of randomization and receive OFA and OBA respectively. The methods for induction and maintenance of anesthesia among different groups have been described in detail previously. The systolic blood pressure and heart rate will be maintained within 20% of the baseline during the operation.

Both groups will be ventilated with a tidal volume of 6–8 ml/kg to avoid barotrauma, the respiratory rate is 10–14 times/min, and the positive end-expiratory pressure (PEEP) was 5–10 cmH_2_O to maintain PetCO_2_ 35–45 cmH_2_O. We will record the hemodynamic instability (vasoactive drugs for hypotension or hypertension, atropine for bradycardia, beta-blockers for tachycardia) and treatments.

Postoperatively, extubation under deep anesthesia with the laryngeal mask retained and transfer participants to the PACU. Sugammadex sodium will be given to antagonize muscle relaxation at a dose of 2–4 mg/kg. The laryngeal mask is generally well-tolerated after the participants awake and will be removed after monitoring for 1 h, and the patient will be transferred safely to the ward after continuing monitoring for 1 h.

### Evaluation and follow-up

One day before the operation, each patient will be given a time-listed NRS form and detailed instructions on how to record score of quiet NRS score and cough NRS score at the different postoperative times (0.5, 1, 2, 3, 7, 11, 15, 19, 23, 27 h, postoperatively) (Showed in Table 2).

Relevant data of participants will be collected by independent researchers. Standardized data collection files (case report forms) will be used to ensure that the data are recorded and used for future statistical analysis. Data collects as follow: gender, age, weight, BMI, polysomnography test results, neck circumference, modified Mallampati score, upper lip bite test, operation time, anesthesia time, days of hospitalization, days of chest drainage, post-operative evaluation, complications, side effects (respiratory depression, hypotension, vomiting, nausea, itching).

### Adverse events

1. Tachycardia: When heart rate > 100 beats/min, or increases by more than 20% from baseline if the baseline value is >83 beats/min, esmolol 10 mg will be given and/or adjust the dose of anesthetics.
2. Hypertension: systolic blood pressure >160 mmHg, or increases from baseline 20% or more if the baseline value >133 mmHg, urapidil 10 mg will be given and/or adjust the dose of anesthetics.
3. Bradycardia: Heart rate < 55 beats/min, or reduces by more than 20% from baseline or if the baseline value is <69 beats/min, atropine 0.3 mg and/or isoproterenol 2 μg or adjust the anesthetics dose.
4. Hypotension: systolic blood pressure < 95 mmHg, or drops more than 20% if the baseline value is <119 mmHg, liquid infusion, ephedrine 6 mg or norepinephrine 4 μg and/or anesthetics dose adjustment will be applied.
5. Intraoperative awareness: During general anesthesia and standard treatment, patients can recall intraoperative events.

Safety assessments will include monitoring and recording of all adverse effects and severe adverse effects and regular monitoring of intraoperative and postoperative critical data including type, time, duration, treatment, and sequelae by the attending anesthesiologists until it is completely resolved or treatment is terminated. Before signing the informed consent, patients will be informed of all potential harms before anesthesia, including the risks of OFA such as oversedation, insufficient analgesia, hallucinations, emotional depression, and severe drug allergy. All adverse effects or possible complications will be compiled in the data collection forms.

If significant risks to patient safety occur during the trial, we will report it to the research group and the ethics committee to evaluate whether the trial should be continued. Appropriate actions, including medical attention, will be taken when necessary.

### Data collection, handling, and monitoring

Relevant data of participants will be collected by independent researchers who are unaware of the research intervention (Table 3).

**TABLE 3:** Description of main and secondary variables.

**Primary outcome** Postoperative NRS score (0–10). 0 means absence of pain and 10 is the severest pain imaginable. Postoperative NRS score was recorded every half an hour for the first hour, every hour for the next 2 h, and every 4 h for the next 24 h. **Secondary outcomes** *Range of nalbuphine requirements* 0: no use 1: <10 mg/day 2: 10–20 mg/day 3: More than 20 mg/day *PONV, incidence and severity of PONV* NRS: A 10 cm ruler was used as the scale. One end of the scale was 0, indicating no nausea and vomiting, and the other end was 10, indicating the severest unbearable nausea and vomiting (1–4 as mild, 5–6 as moderate, 7–10 as severe). *Need for rescue antiemetic medication* 1: Yes 2: No *L-SRS* The surgeon will score the quality of the intra-abdominal conditions at 15 min intervals using the L-SRS [see Martini et al. (22) and Boon et al. (23)]. In brief, the L-SRS is a 5-point Likert scale that enables the quantification of surgical conditions in a standardized fashion. The scale runs from 1 to 5: extremely poor (score = 1), poor (=2), acceptable (=3), good (=4), and excellent (=5) surgical working conditions. *Postoperative agitation* Riker Sedation-Agitation Scale (SAS) (Table 4) *Postoperative chills* Wrench classification: Grade 0, no chills; Grade 1, bundles and/or peripheral vasoconstriction and/or peripheral cyanosis, but no fibrillation; Grade 2, *PACU stay time* *EuroQol five-dimensional questionnaire (EQ-5D)* The EQ-5D descriptive system is a preference-based HRQL measure with one question for each of the five dimensions that include mobility, self-care, usual activities, pain/discomfort, and anxiety/depression. *Length of hospital stay (days)* *Intraoperative awareness* 1: Yes 2: No *Hemodynamically unstable treatments*

**TABLE 4:** Riker sedation-agitation scale.

Score	Term	Description
7	Dangerous agitation	Pulling at endotracheal tube, trying to remove catheters, climbing over the bed rail, striking at staff, thrashing side to side
6	Very agitated	Does not calm, despite frequent verbal reminding of limits; requires physical restraints, biting endotracheal tube
5	Agitated	Anxious or mildly agitated, attempting to sit up, calms down to verbal instructions
4	Calm and cooperative	Calm awakens easily, follows commands
3	Sedated	Difficult to arouse; awakens to verbal stimuli or gentle shaking, but drifts off again; follows simple commands
2	Very sedated	Arouses to physical stimuli, but does not communicate or follow commands, may move spontaneously
1	Unable to rouse	Minimal or no response to noxious stimuli, does not communicate or follow commands

Noxious stimuli: Sputum suction or pressure on the eye socket, sternum, or nail bed for 5 s.

### Randomization, blinding, allocation, and concealment

Patients will be randomized using block randomization with four-block length, stratified to minimize bias on the primary outcome measure. Randomization will be performed electronically after the assessment of eligibility. The participants will be blinded to the group allocation in this study.

Surgical staff and researchers responsible for the post-operative follow-up are blind to the randomized allocation of patients.

The anesthesiologists who are responsible for BS surgery will share no information related to patient randomization.

The statistical analysis will be carried out independently by a separately appointed statistician.

### Sample size

Based on the research from Marija toleska (24) (A prospective, single-blind, randomized controlled study of laparoscopic cholecystectomy using opioid anesthesia and opioid-free anesthesia, mainly observed the postoperative VAS score), the average VAS score of opioid-free anesthesia was 3.27 ± 1.7, while the opioid anesthesia in the control group was 5.13 ± 2.7. PASS 15.0 was used to compare the two groups of mean superiority tests. The sample size was calculated by two-sided test and test level (α = 0.05) The ratio was 1:1, and the power (1-β) was 80%, and considering the shedding rate (10%), we need to recruit 48 participants (24 in each group).

### Statistical analysis

All statistical data analyzes will be performed using the SPSS software (IBM SPSS Statistics V.25).

1. The measurement data conforming to the normal distribution are expressed by mean ± standard deviation (x ± s). Methods Repetitive measure analysis of variance (ANOVA) was used in analyzing the repeated measurement data (NRS score) compared within the group. The independent *t*-test or one-way ANOVA are used for inter-group comparison.
2. The measurement data of non-normal distribution are expressed by median (m) and 25th and 75th percentile (P25, p75). Mann-Whitney U test is used for comparison between groups.
3. Categorical variables will be described as counts (percentages) and compared using χ2 analysis or Fisher’s exact test. The overall significance level is set at *p* < 0.05 and Bonferroni correction will be used to control type I errors.
4. Covariance analysis and logistic regression analysis will be introduced into the model to minimize study factors, confounders and their interaction.

## Discussion

Although opioid anesthesia is now the mainstay of anesthesia, there are still many deficiencies in postoperative pain management, especially in the postoperative phase. There are fewer available options for opioids, and their clinical use is often limited by their side effects such as postoperative nausea and vomiting, respiratory depression, and over sedation. Currently, the medical literature supports the use of intravenous lidocaine, ketamine, and dexmedetomidine as a balanced anesthetic modality for perioperative period management to replace or reduce opioids (20, 25–27). To our knowledge, the analgesic efficacy and clinical value of esketamine in morbid obesity patients undergoing BS remain unclear. To explore this issue, we designed this single-center, prospective, randomized, controlled, single-blind study to elucidate the efficacy of the analgesic management of esketamine-based OFA in morbid obesity patients with OSA undergoing BS.

Esketamine, the S (+)- isomer of ketamine, is safer and suitable for induction and maintenance of general anesthesia. It is approved by the FDA in 2019 as the first new class of antidepressants (28–30). Esketamine is theoretically more analgesic, and nonetheless, the actual analgesic effect of esketamine remains controversial (25, 31, 32). Cheng et al. (33) reported that esketaminea (bolus of 0.25 mg/kg, followed by an infusion of 0.125 mg/kg/h until 15 min before the end of the surgical procedure) improved the quality of rehabilitation in patients undergoing video-assisted thoracic surgery (VATS), and also improved postoperative analgesia and postoperative depression. Another study of the effects of esketamine sedation on hydrostatic reduction of intussusception ketamine (34) found insufficient evidence for a higher success rate, lower relapse rate, shorter duration, and shorter hospital stay with esketamine compared with morphine analgesia. For the chronic opioid-dependent population, a perioperative bolus of 0.5 mg/kg of ketamine followed by an infusion of 0.25 mg/kg/h reduces pain and reduces opioid dependence 1 year after spinal surgery (35).

Opioid-free anesthesia is an anesthesia method based on the concept of multi-mode analgesia, using a combination of multiple drugs or technologies to achieve anesthesia and analgesia, reduce sympathetic reflex, obtain stable hemodynamics, good organ perfusion and high-quality anesthesia recovery, and to meet the perioperative analgesia of patients (17, 36). Although there are still some controversies about the wide application of OFA in the clinic (37), we also see that this technology has been widely applied to the clinical practice of BS, general surgery, bone and spinal surgery, cesarean section and other operations (24–26, 38–41). The application of OFA in obesity showed that it is a safe, feasible and well-tolerated therapy, which may offer a novel and well-tolerated treatment in morbid obesity patients (42, 43).

However, our study also remains some limitations. One of the main limitations for the interpretation of results will be the small sample size of the study, especially regarding the multiple outcomes we plan to analyze. Secondly, considering the small overall sample size, the randomization of this study will not be stratified, and there are obvious difficulties in anesthesia for super-obese patients (BMI > 50), and the long extubation time and wake time, which may bias the results of the statistical analysis results. Finally, as the study is single-blind, and the personnel who performed the anesthesia will know the specific grouping situation, some bias on the study results may appear.

## Ethics statement

The studies involving human participants were reviewed and approved by the Ethics Committee of the First Affiliated Hospital of Shandong First Medical University. The patients/participants provided their written informed consent to participate in this study.

## Author contributions

YS and YW were the principal investigators of this study, obtained grant funding, and refined the study protocol. YG and LC participated in the design of the study protocol, drafted the protocol, and wrote the protocol manuscript. ZG, MZ, ML, XG, YL, XZ, and NG assisted in the development and implementation of the study. YS supervised the study. All authors critically reviewed and approved the final manuscript.

## Abbreviations

OFA: opioid-free anesthesia
BS: bariatric surgery
OSA: obstructive sleep apnea
OBA: opioid-based anesthesia
NRS: numeric rating scale
PONV: postoperative nausea and vomiting
BMI: body mass index
PCIA: patient-controlled intravenous analgesia
CRF: case report form
L-SRS: Leiden surgical rating scale
EQ-5D: EuroQol five-dimensional questionnaire.
